# The impact of antiplatelet therapy on pelvic fracture outcomes

**DOI:** 10.4103/0974-2700.76841

**Published:** 2011

**Authors:** Jonathan M Christy, S Peter Stawicki, Amy M Jarvis, David C Evans, Anthony T Gerlach, David E Lindsey, Peggy Rhoades, Melissa L Whitmill, Steven M Steinberg, Laura S Phieffer, Charles H Cook

**Affiliations:** Department of Orthopaedics, Division of Critical Care, Trauma and Burn, The Ohio State University Medical Center, Columbus, OH, USA; 1 Department of Surgery, Division of Critical Care, Trauma and Burn, The Ohio State University Medical Center, Columbus, OH, USA; 2 Department of Pharmacy, The Ohio State University Medical Center, Columbus, OH, USA

**Keywords:** Antiplatelet therapy, injury severity, pelvic fractures, pelvic severity score, trauma outcomes

## Abstract

**Introduction:**

Despite increasing use of antiplatelet agents (APA), little is known regarding the effect of these agents on the orthopedic trauma patient. This study reviews clinical outcomes of patients with pelvic fractures (Pfx) who were using pre-injury APA. Specifically, we focused on the influence of APA on postinjury bleeding, transfusions, and outcomes after Pfx.

**Methods:**

Patients with Pfx admitted during a 37-month period beginning January 2006 were divided into APA and non-APA groups. Pelvic injuries were graded using pelvic fracture severity score (PFSS)–a combination of Young–Burgess (pelvic ring), Letournel–Judet (acetabular), and Denis (sacral fracture) classifications. Other clinical data included demographics, co-morbid conditions, medications, injury severity score (ISS), associated injuries, morbidity/mortality, hemoglobin trends, blood product use, imaging studies, procedures, and resource utilization. Multivariate analyses for predictors of early/late transfusions, pelvic surgery, and mortality were performed.

**Results:**

A total of 109 patients >45 years with Pfx were identified, with 37 using preinjury APA (29 on aspirin [ASA], 8 on clopidogrel, 5 on high-dose/scheduled non-steroidal anti-inflammatory agents [NSAID], and 8 using >1 APAs). Patients in the APA groups were older than patients in the non-APA group (70 vs. 63 years, *P* < 0.01). The two groups were similar in gender distribution, PFSS and ISS. Patients in the APA group had more comorbidities, lower hemoglobin levels at 24 h, and received more packed red blood cell (PRBC) transfusions during the first 24 h of hospitalization (all, *P* < 0.05). There were no differences in platelet or late (>24 h) PRBC transfusions, blood loss/transfusions during pelvic surgery, lengths of stay, post-ED/discharge disposition, or mortality. In multivariate analysis, predictors of early PRBC transfusion included higher ISS/PFSS, pre-injury ASA use, and lower admission hemoglobin (all, *P* < 0.03). Predictors of late PRBC transfusion included the number of complications, gender, PFSS, and any APA use (all, *P* < 0.05). Mortality was associated with pelvic hematoma/contrast extravasation on imaging, number of complications, and higher PFSS/ISS (all, *P* < 0.04).

**Conclusions:**

Results of this study support the contention that preinjury use of APA does not independently affect morbidity or mortality in trauma patients with Pfx. Despite no clinically significant difference in early postinjury blood loss, pre-injury use of APA was associated with increased likelihood of receiving PRBC transfusion within 24 h of admission. Furthermore, multivariate analyses demonstrated that among different APA, only preinjury ASA (vs. clopidogrel or NSAID) was associated with early PRBC transfusions. Late transfusion was associated with the use of any APA, complications, higher PFSS, and need for pelvic surgery.

## INTRODUCTION

Antiplatelet agent (APA) use is becoming more prevalent as our overall population ages.[1] The use of APA has been noted to be especially common in patient groups considered to be at high risk for polypharmacy–those with multiple co-morbid conditions and age >45 years.[2] It is well documented that the use of acetylsalicylic acid (ASA), clopidogrel, high-dose nonsteroidal anti-inflammatory agents (NSAID), and other APA is increasing among middle- and older-aged patients in various clinical settings (i.e., atherosclerotic vascular disease, following percutaneous vascular interventions, chronic maintenance therapy for osteoarthritis and other chronic musculo-skeletal conditions).[1,3–7] Despite the increasing utilization of APA in middle- and older-aged patients, relatively little is known regarding the impact of these agents on trauma outcomes.[8] Clinical reports describing the relationship between various APA, blood loss, and transfusion requirements in both the general orthopedic and trauma orthopedic literature are few, limited in scope and/or size, and varied in results.[9–15] Controversies continue over the optimal perioperative approach to orthopedic patients on APA, with little to no consensus between orthopedic providers.[9,16] The authors of this manuscript postulate that many orthopedic trauma patients who use pre-injury APA receive unnecessary blood and blood product transfusions, often guided by the belief that APA use predisposes to significant increases in postinjury blood loss. The primary purpose of this study was to better quantify the impact of APA on postinjury bleeding, transfusions, and outcomes in patients with pelvic fractures (Pfx).

## METHODS

This is a retrospective review of consecutive patients >45 years of age with Pfx identified from an American College of Surgeons verified level I trauma center database during a 37-month period beginning January 1, 2006. Inclusion criteria included adult patients ≥45 years of age, who sustained Pfx. Pfx was defined as any combination of the following findings: (a) disruption of the pelvic ring; (b) acetabular fracture(s); and (c) sacral fracture(s). Exclusion criteria included age <45 years, pregnant patients, and prisoners. Of note, we chose to examine patients aged ≥45 years, because the reported use of antiplatelet agents is low in younger patients.[2,17–20]

We defined preinjury antiplatelet therapy as: (a) regular daily use of at least 81 mg of acetylsalicylic acid (aspirin or ASA); (b) regular daily use of at least 75 mg of clopidogrel; (c) scheduled use of prescription-strength nonsteroidal anti-inflammatory drugs (NSAID)–i.e., ibuprofen, naproxen, ketoprofen, etc. The latter group consisted exclusively of patients who take NSAID regularly for chronic musculo-skeletal inflammatory conditions (i.e., osteoarthritis). Patients were grouped as actively receiving one or more regularly scheduled therapeutic APA preinjury or not receiving preinjury antiplatelet therapy (non-APA group).

The following additional clinical variables were collected and analyzed: (a) patient demographics; (b) Injury Severity Score (ISS); (c) Glasgow Coma Scale (GCS); (d) associated injuries; (e) pre-existing medical/co-morbid conditions–hypertension, coronary arterial disease, diabetes, obstructive pulmonary disease, among others; (f) preinjury medications; (g) transfusion of blood (packed red blood cells [PRBC]) and blood product (platelets, fresh frozen plasma [FFP], and cryoprecipitate); (h) hospital and intensive care unit (ICU) lengths of stay (LOS); and (i) laboratory determinations–hemoglobin, hematocrit, prothrombin time (PT), activated partial thromboplastin time (aPTT), and the international normalized ratio (INR).

In order to standardize the classification of pelvic injury severity, we devised a pelvic fracture severity score (PFSS) based on the type(s) of component Pfx involved in each individual case. We combined three different Pfx classifications (Denis, Letournel/Judet, and Young/Burgess), with the presence of hematoma and/or active contrast extravasation (“blush”) on initial pelvic computed tomographic (CT) imaging, as well as the presence of open fracture [Table 1].[21–25] The Tile classification was not utilized because of its significant overlap with the Young and Burgess classification, and thus the potential for introduction of bias.[24] Recognizing that double-column acetabular fractures are often associated with more severe injury mechanisms, extraseverity points were added for that occurrence.

**Table 1:** Details of the pelvic fracture severity scoring system

			PFSS points
1. Denis classification[21]	Type 1	1	
	Type 2	2	
	Type 3	3	Score ____
2. Letournel and Judet classification[22,23]	Any type (AC, AW, PC, PW, TRV)	1	
	Double column *(add extra points)*	3	Score ____
3. Young and Burgess classification[25]	APC1	1	
	APC2	2	
	APC3	3	
	LC1	1	
	LC2	2	
	LC3	3	
	VS	3	Score ____
4. Additional pelvic fracture details	Open fracture	10	
	Hematoma/“Blush” on imaging	20	Score ____
5. Calculated total PFSS	Add components from 1 to 4		Total ____

PFSS - PELVIC FRACTURE SEVERITY SCORING

Hemoglobin and hematocrit data were recorded on admission, and during the 24 h post-admission. In addition, the nadir hemoglobin value within the first 7 days of hospitalization was recorded. The difference between the admission hemoglobin and the lowest pretransfusion hemoglobin during the first 24 h postadmission was calculated and labeled as uncorrected initial hemoglobin drop. In addition, we calculated a corrected hemoglobin drop, which was defined as uncorrected hemoglobin plus 1 g of hemoglobin per each PRBC unit transfused. Lastly, the difference between the initial hemoglobin and the lowest hemoglobin obtained during the period starting 24 h after admission and ending 7 days after admission was recorded and termed 1-week hemoglobin drop.

Data were analyzed using SPSS™ 18 for Windows software (SPSS, Inc., Chicago, IL). Categorical variables were analyzed using *χ*^2^ or Fisher’s exact testing, as appropriate. Continuous variables were analyzed using Student’s *t*-test or analysis-of-variance (ANOVA) methodology. Multivariate analyses were performed utilizing multiple logistic regression for variables with statistical *P* < 0.20 in univariate analyses, and reported as P value and the Wald statistic. Statistical significance was set at α = 0.05. Institutional review board approval was obtained for this study.

## RESULTS

Of 109 patients ages 45 years and older with Pfx, 37 were taking APA at the time of injury. Of these, 29 (78.4%) were taking ASA, and 8 (21.6%) were taking clopidogrel. Five (13.5%) were on high-dose scheduled NSAID. Eight patients (21.6%) were taking more than one APA. In addition, four patients in the ASA sub-group were also taking warfarin, with one patient having nontherapeutic INR and the other three having therapeutic (≥2.0) INR. Interestingly, none of the four patients receiving combined ASA and warfarin had hemorrhagic complications or required any blood or blood product transfusions.

Following initial trauma evaluations, 70/109 (64.2%) patients were admitted to a trauma/orthopedic floor, 26/109 (23.9%) were admitted to surgical ICU, and 13/109 (11.9%) were taken directly to the operating room (for various orthopedic and non-orthopedic indications). None of the patients died in the emergency department. On hospital discharge, of the 97 surviving patients, 29 (29.9%) went home, 38 (39.2%) required rehabilitation facility admission, and 30 (30.9%) were transferred to skilled nursing facilities.

Patients in the APA group were significantly older than patients in the non-APA group (70.1 ± 12.6 vs. 63.0 ± 13.6 years, *P* < 0.01). The APA and non-APA groups were similar in gender distribution, ISS/PFSS, post-ED destination, hospital and ICU lengths of stay, complications, and mortality [Table 2]. Of note, we observed significant differences in the number of preinjury medications and comorbidities between the two groups, with APA group patients taking significantly more preinjury medications and had significantly more comorbid conditions [Table 2]. Despite that, we found no significant differences in standardized patient functional assessments of locomotion, expression-communication, and feeding between the two groups, as recorded on hospital discharge.

**Table 2:** Comparison of general patient characteristics between APA and non-APA groups

	APA *n* = 37	Non-APA *n* = 72	*P* value
Patient age	70.1 ± 12.6	63.0 ± 13.6	0.010
Injury severity score	13.6 ± 12.1	16.7 ± 15.7	0.299
Number of co-morbid conditions	4.54 ± 3.23	1.95 ± 1.72	<0.001
Number of pre-injury medications	6.42 ± 3.77	2.68 ± 1.88	<0.001
Hospital length of stay	8.44 ± 10.2	11.6 ± 20.8	0.399
Intensive care unit length of stay	3.14 ± 9.14	4.51 ± 16.3	0.643
Number of associated non-PFx injuries	3.72 ± 3.38	3.22 ± 3.45	0.477
Pelvic fracture severity score	4.97 ± 7.62	5.74 ± 8.21	0.642
Packed red blood cell (PRBC) transfusion <24 hrs	14/37	12/72	0.018
Platelet transfusion <24 h	4/37	3/72	0.225
Initial admission hemoglobin	12.0 ± 1.65	12.3 ± 2.17	0.435
Hemoglobin 12–24 h post-admission	10.4 ± 1.90	11.3 ± 1.90	0.043
Hemoglobin drop <24 h uncorrected	-1.68 ± 1.15	-1.24 ± 1.22	0.101
Hemoglobin drop <24 h PRBC-corrected	-2.86 ± 3.56	-2.18 ± 3.50	0.376
Hematoma/contrast extravasation on initial CT	7/37	13/72	0.682
Requirement for interventional embolization	2/37	2/72	0.603
Number of complications	0.78 ± 1.38	0.63 ± 1.12	0.537
Overall mortality	4/37	8/72	0.998

Despite no overall differences in uncorrected (1.68 vs. 1.24 g/dL, *P* > 0.05) and PRBC-corrected (2.86 vs. 2.18 g/dL, *P* > 0.05) initial hemoglobin drops, patients in the APA group were significantly more likely to receive a PRBC transfusion (37.8% vs. 16.7%, *P* < 0.02) during the first 24 h of hospitalization [Table 2]. In contrast, there were no differences in platelet, FFP, cryoprecipitate, or late (>24 h after admission) PRBC transfusions between APA and non-APA patients (all, *P* > 0.05). There was no difference between the initial hemoglobin (12.0 g/dL vs. 12.3 g/dL) or the 1-week hemoglobin drop (APA 3.23 g/dL vs. non-APA 2.71 g/dL, *P* > 0.05) between the two groups. Review of operative blood loss and transfusion utilization for procedures involving open pelvic fixation showed that the APA and non-APA groups had similar blood losses (392.86 ± 361.05 mL vs. 452.4 ± 502.3 mL, *P* > 0.05) and intraoperative PRBC transfusions (0.675 ± 0.963 units vs. 0.728 ± 1.132 units, *P* > 0.05). It is important to note that the average time to pelvic operation was not significantly different between the two groups (5.63 ± 5.53 days for the APA group vs. 4.51 ± 5.11 days for the non-APA group, *P* > 0.05).

In univariate analysis, the presence of hematoma/contrast extravasation on initial CT imaging, lower initial hemoglobin, higher ISS, PFSS, aPTT, INR, increasing number of associated injuries, and the use of ASA were all significantly associated with early (<24 h) PRBC transfusion requirement [Table 3]. In multivariate analysis, lower initial (hospital admission) hemoglobin (10.5 g/dL vs. 12.4 g/dL), higher ISS/PFSS, and the use of ASA (44.8% in ASA group received transfusion vs. 9.9% in non-ASA group) were significantly associated with early PRBC transfusion [Table 3].

**Table 3:** Multivariate analyses of factors associated with early (<24 h) packed red blood cell (PRBC) transfusions, late (>24 h) PRBC transfusions, open pelvic surgery, and mortality

Parameter	Predictors of early (<24 h) PRBC transfusion		Predictors of late (beyond 24 h) PRBC transfusion		Factors associated with open pelvic surgery		Predictors of mortality	
Class: Sub-grouping	Univariate, *P*-value	Multivariate, *P*-value (Wald statistic)	Univariate, *P*-value	Multivariate, *P*-value, (Wald statistic)	Univariate, *P*-value	Multivariate, *P*-value (Wald statistic)	Univariate, *P*-value	Multivariate, *P*-value (Wald statistic)
Demographic: Age	0.204		0.011		<0.001	0.004 (8.12)	0.062	
Demographic: Gender	0.064		0.017	0.015 (5.95)	0.005			
Coagulation: INR	0.014						0.001	
Coagulation: PT								
Coagulation: PTT	0.022						0.003	
Hematologic: Any blood products given–Initial 24 h			<0.001		0.484		0.008	
Hematologic: Corrected hemoglobin drop–Initial 24 h			<0.001		0.106		0.109	
Hematologic: Initial hemoglobin	<0.001	0.024 (5.12)	0.164				0.008	
Hematologic: Fresh frozen plasma transfusion >24 h			0.010		0.003			
Hematologic: PRBC transfusion–Initial 24 h			<0.001				<0.001	
Hematologic: PRBC transfusion >24 h					<0.001	<0.001 (12.8)		
Pharmacologic: Any antiplatelet agent use	0.307		0.060	0.008 (7.096)	0.085		0.656	
Pharmacologic: Acetylsalicylic acid use	0.049	0.020 (5.42)					0.915	
Pharmacologic: Clopidogrel use	0.610		0.091					
Pharmacologic: High-dose/scheduled NSAID	0.238							
Metric: Injury severity score	<0.001	0.025 (5.03)	0.027		0.536		<0.001	0.014 (4.28)
Metric: Number of associated non-PFx injuries	<0.001		<0.001		0.154		0.005	
Metric: Number of complications			<0.001	0.003 (10.5)	0.133		<0.001	0.015 (6.68)
Metric: Pelvic fracture severity score	0.001	0.008 (7.003)	<0.001	0.047 (3.94)	0.051	0.010 (6.70)	0.035	0.034 (4.51)
Procedural: Need for IR intervention	0.095		0.238		0.467		0.367	
Procedural: Need for open pelvic surgery			<0.001	0.002 (9.40)			0.029	0.011 (6.82)
Radiographic: Hematoma/contrast extravasation on CT	0.029		0.002		0.156	0.009 (6.89)	0.010	0.027 (4.92)

Factors associated with late (>24 h) PRBC transfusions in univariate analysis included patient age/gender, early (<24 h) blood or blood product transfusion, greater initial PRBC-corrected hemoglobin drop, ISS/PFSS, the number of associated non-Pfx injuries, the number of complications, need for open pelvic surgery, and the presence of hematoma/contrast extravasation on CT scan [Table 3]. Predictors of late PRBC transfusion in multivariate analysis included patient gender, preinjury use of any APA medication, the number of complications, need for open pelvic surgery, and PFSS [Table 3].

Of the 109 patients, 32 (29.4%) required operative intervention for their Pfx. The time between hospital admission and open pelvic intervention was not significantly different between the APA (5.63 ± 5.53 days) and non-APA groups (4.51 ± 5.11 days, *P* > 0.05). In univariate analysis, the need for open operative treatment of Pfx was associated with patient age/gender, and PRBC/FFP transfusion beyond the initial 24 h (all, *P* < 0.01). In multivariate analysis, patient age (58.1 for operative vs. 68.7 for non-operative group), PRBC transfusion beyond the initial 24 h (47% transfused in operative group vs. 11% in nonoperative group), the presence of hematoma/blush on CT, and PFSS were significantly associated with open operative treatment of Pfx (all, *P* < 0.01) [Table 3].

The overall mortality in this study was 12/109 or 11%. Univariate analysis demonstrated that the receipt of early (within 24 h of admission) PRBC/blood products, the number of associated injuries, the presence of pelvic hematoma/contrast extravasation on initial CT imaging, lower initial hemoglobin, elevated aPTT/INR, higher PFSS/ISS, and need for pelvic surgery were all associated with mortality [Tables 3 and 4]. In multivariate analysis, patient mortality was associated with the presence of pelvic hematoma/contrast extravasation on CT (42% in mortality group versus 14% in survivors), greater number of complications (1.83 vs. 0.526), higher PFSS (10.0 vs. 4.88), greater ISS (41.3 vs. 13.1), and need for pelvic surgery (all, *P* < 0.04) [Tables 3 and 4].

**Table 4:** Comparison of general patient characteristics between survivors and non-survivors

	Survivors	Non-survivors	Significance
Number of complications	0.53 ± 1.05	1.83 ± 1.70	*P* < 0.001
Injury severity score	31.1 ± 10.9	41.3 ± 24.3	*P* < 0.001
Initial hemoglobin	12.4 ± 1.90	10.6 ± 2.39	*P* = 0.008
Initial INR	1.91 ± 0.326	2.78 ± 4.59	*P* = 0.001
Initial PTT	29.5 ± 7.68	47.7 ± 50.0	*P* = 0.003
Number of associated injuries	2.76 ± 3.11	5.67 ± 4.72	*P* = 0.005
Pelvic fracture severity score	4.88 ± 7.48	10.0 ± 10.5	*P* = 0.035

## DISCUSSION

This study shows that while preinjury use of APA is not associated with significantly worse bleeding after Pfx, patients receiving preinjury APA were more likely to receive PRBC transfusions early during their hospital admissions. Although it is difficult to determine the precise reason for this observation due to the retrospective nature of this study, one could speculate that this finding could reflect the prevailing belief by some practitioners that APA use may contribute to worse early postinjury bleeding. While others reported that patients who use APA tend to have lower initial hemoglobin concentrations,[13] our study shows that the admission hemoglobin concentrations did not differ between the APA and non-APA groups [Table 2]. Some authors note increased perioperative blood loss in patients taking APA before orthopedic procedures,[9,26] while others have reported no effect.[5,13] In this study, there were no statistical differences in terms of operative blood loss or blood transfusions between APA and non-APA groups. However, due to an average operative delay of several days, as well as our practice pattern of discontinuing APA use after trauma admission, it is unlikely that APA use contributed significantly to operative blood loss or blood product administration in this study.

With regards to blood transfusions, Manning *et al*. showed that patients taking APA were more likely to receive postoperative PRBC transfusions.[13] This is also consistent with observations that total hip arthroplasty patients using preoperative ASA or NSAID had greater intraoperative and postoperative blood loss when compared to patients not taking APA.[26] While we noted increased early (<24 h) incidence of PRBC transfusions, our data show no significant differences between APA and non-APA groups for PRBC transfusions after the initial 24 h, operative blood loss, or operative PRBC administration. Considering the pelvic operative delay among study patients, along with the “fading” therapeutic effect of APA, the association between late (>24 h after admission) PRBC transfusion and pelvic surgery seems to be predominantly due to perioperative and operative factors, and not residual effects of APA.

It is interesting, and perhaps even counterintuitive that ASA, and not clopidogrel was associated with increased requirement for early PRBC transfusions. Data on the effects of clopidogrel in orthopedic practice are still limited, and there is lack of much needed consensus regarding best clinical practices in this setting.[7,16] Johansen *et al*. reported that clopidogrel contributes to perioperative blood loss, and that this contribution is reduced by postponing surgery for at least 5 days (coincidentally similar to the average pelvic operative delay in this study).[27] Lavelle *et al*. suggested that a perioperative platelet transfusion may offer some benefit to operative hemostasis in patients taking preoperative clopidogrel.[16] Same authors also advocated that early operative intervention (within 2 days of admission) may still be in the patient’s best interest despite the possibility of increased blood loss.[16] Yet others suggest that stopping clopidogrel 1 week prior to surgery in elective cases, and stopping clopidogrel on admission in emergent cases constitutes the most optimal approach.[7] Highlighting the expected increase in the use of clopidogrel in the older patient population, Inman *et al*. recommend that institutional protocols for perioperative withdrawal and resumption of clopidogrel may be of benefit when treating this growing segment of patient population.[8] In the current report, the mean time between hospital admission and open pelvic intervention was 5.6 days in the APA group and 4.5 days in the non-APA group, which was not significantly different. Of note, it is our pattern of practice, unless absolutely contraindicated, to discontinue ASA and clopidogrel use following trauma admission.

Kennedy *et al*. reported that the fracture site, rather than the preoperative use of ASA, was predictive of blood loss and transfusion requirement in patients with hip fractures.[11] However, others suggest that ongoing APA therapy prior to elective major orthopedic surgery significantly increases perioperative blood loss.[14,26,27] Although reports of APA-associated blood loss are common, such bleeding has not been conclusively shown to be of clinical significance.[9] In terms of early PRBC transfusions, we found that both pre-hospital ASA use and higher PFSS were significant contributors in multivariate analysis [Table 3]. For late PRBC transfusions, any APA use along with PFSS contributed significantly [Table 3]. Given the lack of differences in early hemoglobin drop in this study, along with the observed pelvic operative delays and associated “fading” of the APA therapeutic effect, additional clinical investigation is needed to better define any interplay between both injury- and APA-related factors and to build upon the results of our multivariate analyses.

Approximately one-third of patients in our study required operative intervention for their Pfx. In multivariate analysis, younger patient age, PRBC transfusion beyond the initial 24 h, the presence of hematoma/blush on CT, and higher PFSS were all significantly associated with open pelvic surgery. As mentioned previously in this discussion, the relationship between PRBC transfusion and operative Pfx management is most likely due to perioperative and operative factors, and does not seem to be directly related to APA use. The association of younger age and open operative intervention in this study may be explained by the observation that younger (<75 years old) patients had higher PFSS than older (>75 years old) patients.

Our data support the contention that use of APA in the setting of modern orthopedic trauma environment does not seem to affect morbidity or mortality among patients with Pfx.[9] The overall mortality in this study was 11% and did not significantly differ between APA and non-APA groups. Variables independently associated with mortality included the presence of pelvic hematoma/contrast extravasation on initial CT imaging, the number of complications, and higher PFSS/ISS. These observations are consistent with previously published data, which also point to active pelvic bleeding, increasing severity of Pfx, the presence of complications, and overall injury severity as contributory to mortality among patients with Pfx.[28–31]

This study provides an acceptable comparison platform to other studies in terms of overall gender distribution.[5,12–14] With regards to patient age, our patient sample was similar to those of Slappendel *et al*.[14] and Dahl *et al*.[5] but younger than that in studies performed by Foss *et al*.[12] and Manning *et al*.[13] Limitations of this study include its relatively small sample size and inability to determine causal relationships due to the retrospective nature of our analysis. In addition, the lack of similar studies from orthopedic trauma literature limits our ability to compare the current data to previously published studies. Although methodological differences between this study and previously published studies (both from trauma and non-trauma populations) limit the applicability of direct comparisons or ability to draw definitive conclusions, important generalizations can be made after comparing our study and previously published orthopedic literature on this topic.[5,12–14,16,26,27,32] It is also important to note that, given the average pelvic operative delay in this study, the effect of APA on operative blood loss and transfusion requirements in our patient sample is likely negligible. The authors fully recognize that, given the fact that significant proportion of sacral fractures occur within the context of pelvic ring injury, there is potential for an overlap between Pfx classifications within the PFSS.[21–25] The traditional use of multiple different classifications for Pfx description makes interstudy comparisons difficult, highlighting the need for an objective, standardized system of Pfx severity assessment. Although not validated, and designed specifically to help this study’s authors more objectively compare APA and non-APA groups with regards to overall Pfx severity, this proposed score represents an attempt to design such an objective, standardized method of estimating Pfx severity.

## CONCLUSIONS

Despite the lack of differences in hemoglobin concentration drops between APA and non-APA groups, we found that the use of preinjury APA was associated with increased likelihood of receiving PRBC transfusion early after injury. The use of APA did not appear to significantly affect overall complications or mortality. Of interest, multivariate analysis shows that only preinjury ASA use, and not other APA use, is predictive of early postinjury transfusion. Also in multivariate analysis, lower initial hemoglobin levels and greater number of associated injuries were found to be predictive of early transfusion. Predictors of mortality included the presence of a pelvic hematoma/contrast extravasation on CT imaging, increasing number of complications, PFSS, and ISS. Further prospective validation of the PFSS is warranted.
